# Development of a direct competitive ELISA for the detection of *Mycoplasma bovis* infection based on a monoclonal antibody of P48 protein

**DOI:** 10.1186/1746-6148-10-42

**Published:** 2014-02-18

**Authors:** Ping Fu, Zhenhong Sun, Yuewei Zhang, Ziqiang Yu, Haiyan Zhang, Dan Su, Fei Jiang, Wenxue Wu

**Affiliations:** 1 Key Laboratory of Animal Epidemiology and Zoonosis, Ministry of Agriculture, College of Veterinary Medicine, China Agricultural University, Beijing, China; 2 Shandong Vocational Animal Science and Veterinary College, Weifang, China

**Keywords:** Direct competitive ELISA, *Mycoplasma bovis*, Monoclonal antibody

## Abstract

**Background:**

*Mycoplasma bovis* (*M. bovis*) is a major, but often overlooked, pathogen documented to cause respiratory disease, mastitis, and arthritis in cattle throughout China since 2008. Here, we report the development of a direct competitive enzyme-linked immunosorbent assay (Dc-ELISA) to detect *M. bovis* antibody.

**Results:**

We used a recombinant P48 protein and monoclonal antibody (mAb) 10E. MAb 10E, prepared against the recombinant P48 protein of *M. bovis*, identified all *M. bovis* strains with no cross-reactivity with other related pathogens. Coating micro plates with P48 protein instead of whole *M. bovis* cells as well as the use of mAb 10E produced a specific and sensitive Dc-ELISA for *M. bovis* antibody detection with a cut-off percent inhibition (PI) value of 32%. Compared with two commercial indirect ELISA (i-ELISA) kits, our Dc-ELISA offered a higher positive detection rate in 165 clinical bovine serum samples.

**Conclusions:**

A rapid, sensitive, and reliable serological diagnosis method was developed for *M. bovis*, which can facilitate *M. bovis* surveillance, assisting researchers in understanding the ecology and epidemiology of *M. bovis*.

## Background

*M. bovis* is a major, but often overlooked, pathogen causing respiratory disease, mastitis, and arthritis in cattle. First isolated from the milk of mastitic cow in 1961 in US, this bacteria appears to have spread across Ireland, South America, and Europe [1]. In China, the first *M. bovis* pneumonia case was reported in Hubei province in 2008, and the pathogen genome was sequenced in 2011 [2,3]. Until now, *M. bovis* pneumonia cases, mostly in cattle, have been reported in more than ten Chinese provinces. And it often outbreaks in calves after transport. The mAbs used for *M. bovis* detection were reported previously, and most of them were selected against whole *M. bovis* cells [4,5] or *M. bovis* variable surface lipoproteins (Vsps) that undergo high-frequency phase and size variations [6].

P48 protein is an immunodominant invariable lipoprotein exposed on the membrane of *M. agalactiae*[7]. The *M. bovis* P48 protein is homologous to that of *M. agalactiae*, which was first studied in 2005. A single protein band was observed in rabbit antiserum raised against P48 protein of *M. agalactiae* in a *M. bovis* reference strain PG45 and 10 field isolates. An Antibody response against *M. bovis* recombinant P48 protein has been detected both in experimentally and naturally infected animals, suggesting a stable expression of the corresponding genes. Over all, P48 protein is a useful marker for *M. bovis* infection and an alternative candidate for the development of specific serological test for *M. bovis*[8].

Here, we developed a direct competitive ELISA (Dc-ELISA) to detect *M. bovis* specific antibody in serum based on a P48 protein mAb 10E. This Dc-ELISA showed a higher positive rate than two commercial i-ELISA kits in detection of 165 Chinese clinical bovine serum samples.

## Results

### Expression of P48 protein in *E. coli*

**Figure 1:**
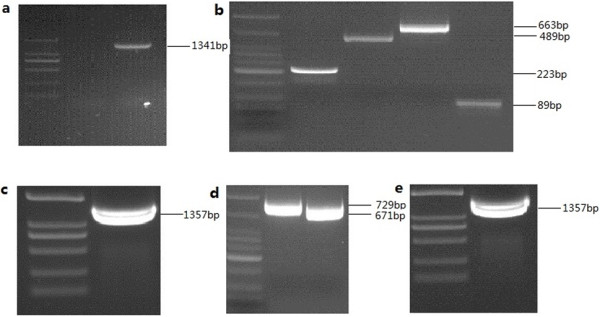
**Identification and mutation of the P48 gene in *M. bovis *strain Hubei-1. a**: Identification of P48 gene in *M. bovis* strain HB-1, using primer MB-F and MB-R primers. An amplified product of 1,341 bp as expected was obtained. **b**: Mutation of the bases on 192, 648, 1,275, 1,317 in the first PCR run. Four overlapping fragments, as expected, were 223 bp, 489 bp, 663 bp and 89 bp. **d**: Mutation of the base on 692 in the third PCR run, two overlapping fragments, 729 bp and 671 bp as expected were obtained. **c** and **e**: full length product obtained using purified fragments as template (MB-a and Mb-d’ primer) in the second and fourth PCR runs, respectively.

**Figure 2:**
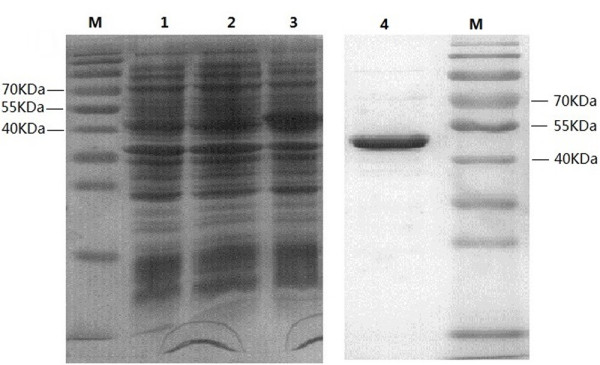
**SDS-PAGE showing expression and purification of P48 in *E. coli*.** All protein was subjected to 8-12% SDS-PAGE analysis and the gel was stained with Coomassie blue. M: prestained protein molecular weight marker (10-170 KDa), lane 1-3: total cell lysates of Transetta (DE3), Transetta (DE3) with PET28 (a+) plasmid, Transetta (DE3) with PET28 (a+)-P48 gene plasmid. Proteins were extracted after 1 mM IPTG induction for 6 h. Lane 4: purified P48 protein using His · Bind kits. Approximately 50 kDa protein band with a purity of 95% was observed.

To clone P48 gene without leader peptide of *M. bovis* strain Hubei-1, a pair of primers (MB-F, MB-R) was used. The amplified 1,341 bp product was cloned and sequenced, and data showed that five UGA codons were present (192, 648, 699, 1,275, 1,317) in the target gene. Therefore, five pairs of mutagenic primers were designed to mutate UGA into UGG using overlap extension PCR with some modifications. In the first and second PCR runs, the bases on 192, 648, 1,275, 1,317 were mutated from “A” to “G”, with the 5′ and 3′-terminus joined fragments bearing restriction sites for Nco I and Xho I to facilitate cloning. After the third and fourth runs, the base on 699 was mutated from “A” to “G” (Figure 1). The mutated P48-lacking peptide gene was then cloned into plasmid PET28 (a+) and transferred into Transetta (DE3). Approximately a 50 kDa protein band was was analyzed by SDS-PAGE (Figure 2).

### Selection and characterization of mAb against P48 protein

To select mAbs with the desired affinity, hybridomas were screened by i-ELISA, and positive cells were selected and subcloned at least three times using a limiting dilution method. MAbs isotypes, detected using the commercial ELISA kit protocol, were all lgG1 (data not shown). One monoclone (from 6 monoclones) designated 10E, which could produce an antibody that specifically recognized P48 protein, was selected to produce and purify mAb from mouse ascetic fluid of mice for further characterization.

**Figure 3:**
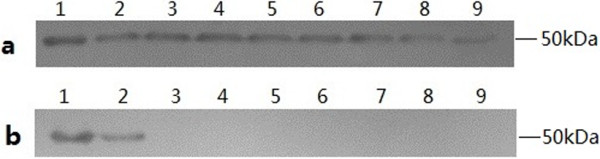
**Western blotting performed to investigate reactivity of mAb 10E with different pathogens. a**: Lane 1-9 respectively was: purified P48 protein, *M. bovis* strain PG45, Hubei-1 SD-2, GY-2, PD-2, HRB-1, HY-1, HG-1. **b**: Lane 1-9 respectively was: purified P48 protein, *M. bovis* strain PG45, *M. agalactiae*, *M. bovirhinis*, *M. arginie*, IBRV, BADV3, BPIV3, BVDV.

MAb 10E was used to investigate the reactivity with different pathogens by Western blot. As shown in Figure 3, mAb 10E reacted with molecular weight of 50 kDa in all eight *M. bovis* strains, but did not react with the other related pathogens including *M. agalactiae*. Homology analysis using the NCBI BLAST server indicated that P48 protein amino acid sequences of *M. bovis* strain Hubei-1 and *M. agalactiae* were similar (74%). Also mAb 10E was not cross-reactive with *M. agalactiae*, likely due to the differences in amino acid sequences or in antibody-binding sites between P48 proteins of *M. agalactiae* and *M. bovis*. This suggests that mAb 10E was specific, not only to P48 protein, but also to *M. bovis*.

### Determination of cut-off value and specificity of Dc-ELISA

**Figure 4:**
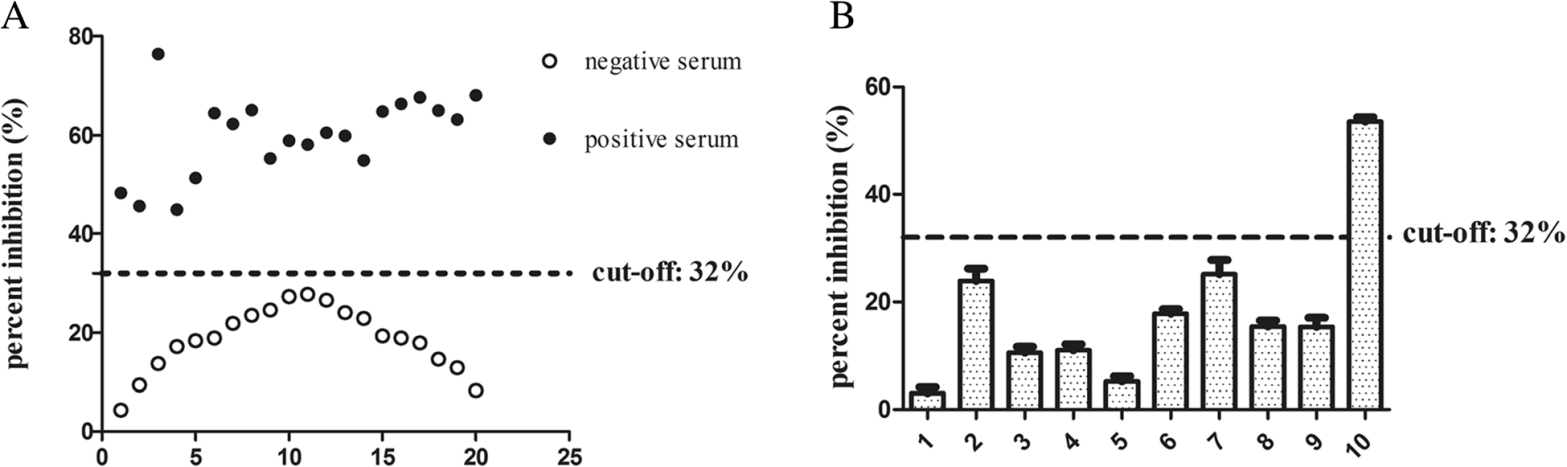
**Establishment of Dc-ELISA. (A)** Distribution of inhibition values of 20 negative and 20 positive serum samples by Dc-ELISA. A cut-off value was set as 32% (mean + 2SD). Positive sera had PI values exceeding 32% and negative sera had PI values less than 32%. **(B)** Inhibition of binding of P48 protein-specific mAb 10E binding by antisera against various pathogenic species. Lanes 1-10 respectively are: BADV3, BVDV, BPIV3, BRSV positive control serum, Rabbit hyperimmune antisera against *M. bovirhinis*, *M. arginine* and *M. agalactiae*, rabbit negative serum, bovine negative *M. bovis* serum, bovine *M. bovis* positive serum. Only the *M. bovis* positive serum had clear inhibition of mAb binding. The Error bars indicate the standard deviations from three-well replications for each serum sample.

After protocol optimization, 20 negative bovine sera and 20 positive bovine sera were used to determine the cut-off value. Because the PI p-value of negative sera as analyzed by the Shapiro-Wilk test was 0.475, we concluded that the data were normally distributed with a mean percent inhibition of 20.78% (SD = 5.69%). Hence, the cut-off value was set as 32% (mean + 2SD) to determine the status of serum samples in response to *M. bovis* antibodies (Figure 4A). All the positive sera had PI values exceeding 32%, suggesting that the cut-off value of 32% was appropriate for distinguishing between negative and positive serum obviously.

To confirm Dc-ELISA specificity, *Infectious bovine rhinotracheitis virus* (IBRV), *bovine viral diarrhea virus* (BVDV), *bovine parainfluenza virus type 3* (BPIV3), and bovine respiratory syncytial virus (BRSV) positive control serum, rabbit hyperimmune antiserum against *Mycoplasma bovirhinis* (*M. bovirhinis*), *Mycoplasma arginine* (*M. arginine*), and *M. agalactiae* were used in this study. As shown in Figure 4B, a PI level lower than 32% was considered as negative, suggesting that the Dc-ELISA was specific for detecting antibody against *M. bovis*.

### Comparison among Dc-ELISA, two commercial i-ELISA kits, and Dot blot

A total of 165 clinical bovine sera originated from various farms in different provinces were tested by Dc-ELISA. Data were compared with that obtained from two commercial i-ELISA kits and a Dot blot assay. Data appear in Table 1, 70 sera were tested negative and 59 sera tested positive by all four methods. In the other 36 sera, except one negative in Dc-ELISA but positive in Dot blot, 35 sera were confirmed to be positive by Dc-ELISA, and some of them (15 by Biovet kit, 29 by Bio-X kit, 2 by Dot blot) were missed by three methods. There were good agreements between Dc-ELISA and Biovet (kappa = 0.819), Bio-X (kappa = 0.66), Dot blot (kappa = 0.963).

**Table 1:** **Results of 165 bovine serum samples for *M. bovis *** **antibody detection by Dc-ELISA, Biovet, Bio-X and Dot blot**

**Sample number**	**Dc-ELISA**	**Biovet**	**Bio-X**	**Dot blot**
70	-	-	-	-
59	+	+	+	+
9	+	-	-	+
5	+	-	+	+
19	+	+	-	+
1	+	+	+	-
1	+	-	-	-
1	-	-	-	+

-: negative result; +: positive result.

The positive rate of clinical serum samples from five provinces was listed in Table 2. Generally, there was quite a high serological positive rate in all the five provinces by four methods. Especially in Shandong province, the positive rate was 75.8% by Dc-ELISA. All those results showed that there was a relatively wide serological population of *M. bovis* in China.

**Table 2:** Positive rates of serum samples from different provinces in China detected by four methods

**Province**	**Dc-ELISA**	**Biovet**	**Bio-X**	**Dot blot**
Beijing	59.1% (13/22)	50% (11/22)	31.8% (7/22)	59.1% (13/22)
Hebei	53.1% (17/32)	40.6% (13/32)	34.4% (11/32)	53.1% (17/32)
Tianjin	61.9% (13/21)	42.9% (9/21)	42.9% (9/21)	61.9% (13/21)
Shandong	75.8% (25/33))	69.7% (23/33))	63.6% (21/33))	72.7% (24/33))
Heilongjiang	43.9% (25/57)	40.4% (23/57)	31.6% (18/57)	45.6% (26/57)
Total	60.0% (94/165)	47.9% (79/165)	39.4% (65/165)	56.4% (93/165)

## Discussion

A simple, rapid, specific and sensitive diagnostic technique is preferred for intensive surveillance of animal infectious diseases such as *M. bovis*. Fortunately *M. bovis* grows well in a variety of media and produces “centered” colonies within 3-5 days, enabling the culture of these pathogens for study and assay development [9]. Ball reported that a sandwich ELISA using monoclonal antibodies for *M. bovis* antigen has similar sensitivity to that of conventional culture diagnosis [10], and he also reported real-time PCR was better for detecting *M. bovis* than sandwich ELISA, which suggested that real-time PCR could replace the sandwich ELISA for *M. bovis* detection [11]. Conventional PCR assays are developed based the 16S rRNA gene [12,13], but some researchers suggested that the PCR-based *uvrC* gene is more specific [14,15]. *In situ* hybridization and immunohistochemistry [16,17] also have been used to identify *M. bovis*.

Many serological tests including indirect hemagglutination and film inhibition have been reported, but these methods were inferior to i-ELISA using whole cell or chemically treated antigens [1,18,19]. And, i-ELISAs based on recombinant VspA, membrane lipoprotein P48 protein [8,20] or glyceraldehyde-3-phosphate dehydrogenase (GAPDH) [21] have been developed. In a competitive/blocking ELISA, immunoglobulins from positive serum samples compete to inhibit the mAb from binding to its specific binding site on antigens and subsequently prevent color development. In contrast, non-reactive sera produce a strong colored reaction [5]. Competitive/blocking ELISAs have been confirmed to be significantly sensitivity and specific for detecting antibody to *Bluetongsue Virus*[22], *West Nile virus*[23], and *Francisella tularensis*[24], and even a higher sensitivity than i-ELISA for detection to *Mycoplasma meleagridis*[25] and PRRSV [26]. A blocking ELISA with a mAb to a certain antigen and the whole-cell lysate of *M. bovis* as the target antigen is a high specific with a good sensitivity, low background, and no cross-reactivity [5].

Here, we report the development of a Dc-ELISA with a mAb 10E against P48 protein and P48 protein as the coated-antigen. MAb 10E was quite specific for all eight *M. bovis* strains and did not cross-react with other related pathogens as evidenced by Western blot (Figure 3). Thus, we concluded that a Dc-ELISA with micro plates coated with purified recombinant P48 protein was more specific than those coated with whole-cell lysates of *M. bovis*. Antisera of other pathogens had PI values less than 32%, indicating that our Dc-ELISA was more specific for the detection of antibodies against *M. bovis* (Figure 4B). Furthermore, the mAb 10E was conjugated with HRP, which replaced the use of HRP-conjugated goat-anti-bovine IgG and the procedure steps were simplified.

A total of 165 clinical bovine serum samples (Table 2) were assayed, and Dc-ELISA (93/165) detected more samples than a Biovet commercial i-ELISA kit (79/165) or a Bio-X kit (65/165). Data from the Dc-ELISA suggested a relatively wide serological population of *M. bovis* in the studied provinces (more than 43.9% by Dc-ELISA), especially in Shandong province (75.8%). Simultaneously, PCR detections of *M. bovis* has been done with the nasal swab samples from Shandong province using MB-F/R primers. In these 33 samples from Shandong, 25 samples were serologically positive by Dc-ELISA whereas 27 samples were etiologically positive, including all the 25 serologically positive ones (data not shown). These results showed that PCR was more sensitive than Dc-ELISA, a finding that agreed with Ali Ghadersohi’s report [5]. Generally, during early stages of microbiological infection, less antibodies are produced, so PCR pathogen detection is better than serological detection for the evaluation of infection. In contrast Dc-ELISA is reliable when antibiotics have been used to treat an infection and Dc-ELISA is simpler and more suitable for high-throughput detection. The Dc-ELISA method we we developed holds promise for facilitating surveillance of *M. bovis*, ultimately leading to a better understanding of the ecology and epidemiology of *M. bovis*.

## Conclusion

A Dc-ELISA based on mAb 10E, which was prepared against P48 protein and capable of identifying all *M. bovis* strains with no cross-reactivity to related pathogens, was developed to detect *M. bovis* infection. With a PI cut-off value of 32%, the Dc-ELISA had higher positive detection rates than two commercial i-ELISA kits when they were used to detect 165 clinical bovine serum samples, which suggested that Dc-ELISA represented an improved and simplified method for surveillance of *M. bovis*.

## Methods

### Mycoplasma and virus strains

Mycoplasma and Viral strains used in this study are listed in Table 3. *M. bovis*, *M. bovirhinis*, *M. arginine*, and *M. agalactiae* were grown in modified PPLO medium containing 20% horse serum. IBRV, *Bovine adenovirus type 3* (BADV3), BVDV, and BPIV3 were cultured in MDBK cells.

**Table 3:** Various Mycoplasma and Virus species used in this study

**Strain**	**Source**
*M. bovis*	ATCC25523(PG45)^a^
*M. bovis*	Hubei-1^b^
*M. bovis*	SD-2^b^
*M. bovis*	Field isolates (GY-2, PD-2, HRB-1, HY-1, HG-1)^c^
*M. bovirhinis*	ATCC27748(PG43)^a^
*M. arginine*	CVCC346(G230)^d^
*M. agalactiae*	CVCC344(PG2)^d^
IBRV	CVCC AV346^d^
BVDV	CVCC^d^
BADV3	ATCC VR-639^e^
BPIV3	ATCC VR-281^e^

^a^American type culture collection, America.

^b^China animal health and epidemiology center, Qingdao, China.

^c^Field isolated from lungs or nasal swabs of bovines having clinical symptoms of *M. bovis* in China.

^d^China veterinary culture collection center, Beijing, China.

^e^China center for type culture collection, Wuhan, China.

### Serum samples

Twenty negative bovine serum samples were collected from cattle without a history of *M. bovis* infection. Twenty positive bovine serum samples were collected from naturally infected *M. bovis* cattle, from which *M. bovis* had been isolated nasal swabs using PPLO medium. Bovine BADV3, IBRV, BVDV, and BPIV3 positive control sera were purchased from Real Bio-technology (Qingdao, China). Rabbit hyperimmune antisera against *M. bovirhinis*, *M. arginine* and *M. agalactiae* were prepared as per standard techniques [27]. Here 165 clinical bovine serum samples were collected from five provinces in China (Beijing, Hebei, Tianjin, Shandong and Heilongjiang) and stored at -20°C.

### Preparation of recombinant P48 protein

To clone the P48 protein encoding gene of of *M. bovis* strain Hubei-1, a pair of primers, MB-F and MB-R, were designed based on the full-length gene (Genbank AY557344.1) without a leader peptide gene [8]. Amplified products were cloned into pEasy-Blunt plasmid (Transgen, Beijing, China). Sequence analysis results indicated that the *M. bovis* P48 gene contained five UGA codons, which is treated as tryptophan according to mycoplasma genetic code, but interpreted as a stop codon in the *E. coli* expression system. To avoid expression of truncated gene products, five pairs of mutagenic primers (shown in Table 4) were designed to mutate UGA into UGG. There were four overlapping PCR runs [28]. In the first PCR run, four amplified products were obtained by using primers MB-a/a’, MB-b/b’, MB-c/c’, MB-d/d’ respectively in four tubes. In the second PCR run, MB-a/d’ were used as primers and the four amplified products were used as the target DNA, therefore, the bases on 192, 648, 1,275, 1,317 were mutated from “A” to “G”, with the 5′ and 3′-terminal joined fragments bearing restriction sites for Nco I and Xho I. In the third PCR run, primers MB-a/e’ and MB-f/d’ were used in two tubes (using the amplified products obtained in the second PCR run as the target DNA) to obtain two amplified products. In the fourth run, MB-a/d’ were used as primers and the two amplified products were used as the target DNA. After the fourth runs, the base on 699 was mutated from “A” to “G”. All the PCR runs were performed as below: 5 min at 94°C, then 20 s at 94°C, 20 s at 61°C, and 30 s at 72°C, 30 cycles, followed by 5 min at 72°C.

**Table 4:** **Primer sets for identification and mutation of P48 gene in *M. bovis *** **strain Hubei-1**

**Primer**	**Sequence (5′–3′)**	**Aim**
MB-F	GCTTCATGTGGTGATAAATACTTTA	Identify P48 gene
MB-R	CTATTTTTGTGTTTCTTTAGCCAAT	
MB-a	CATGCCATGGGCGCTTCATGTGGTGATAAATACTTTA	Mutate point 192
MB-a’	TGAAATTTTATGAACTGCTTCCCAACCAGATTGATTG	
MB-b	TCAATCAATCTGGTTGGGAAGCAGTTCATAAAATTTC	Mutate point 648
MB-b’	CAGAAACTGCTGGCCAAGGAATACCACCGAAGG	
MB-c	CCTTCGGTGGTATTCCTTGGCCAGCAGTTTCTG	Mutate point 1275
MB-c’	CAGCAGGTTGGTCTGGCCATTTTGTACCTAAAGC	
MB-d	CTTTAGGTACAAAATGGCCAGACCAACCTGCTGACC	Mutate point 1317
MB-d’	CTATTTTTGTGTTTCTTTAGCCAACCAGTTAATCATTTTC	
MB-e’	GCTTCTGGGTGTTCTTTATTCCAGTCAATAATACCTTGG	Mutate point 699
MB-f	CATTCCAAGGTATTATTGACTGGAATAAAGAACACCC	

The mutated gene was cloned into the pET28a (+) expression vector. Transetta (DE3) Chemically Competent Cell (Transgen, Beijing, China) was used to express P48 protein. Total cell lysates were obtained by sonicating, and P48 protein was recovered from the soluble fraction and purified using His · Bind kits (Novagen, Darmstadt, Germany). Purity and yield of the recombinant P48 protein was evaluated by SDS-PAGE and the Bradford method [29].

### Preparation of MAb against P48 protein

Female BALB/c mice (8 weeks-of-age) were purchased from the Meiliyaweitong Experimental Animal Technology (Beijing, China). All animal research was approved by the Beijing Association for Science and Technology (approval ID SYXK (Beijing) 2007-0023) and complied with the guidelines of Beijing Laboratory Animal Welfare and the Ethics of the Beijing Administration Committee of Laboratory Animals. All animal studies were also performed in accordance with the China Agricultural University Institutional Animal Care and Use Committee guidelines (ID: SKLAB-B-2010-003) and approved by the Animal Welfare Committee of China Agricultural University. The mice were first inoculated subcutaneously(sc) with 100 μg of purified P48 protein particles in complete Freund’s adjuvant and three weeks later, animals were immunologically boosted with the same dose of P48 protein (sc injection) in incomplete Freund’s adjuvant. Another boost was administered with 25 μg (sc) of P48 protein in incomplete adjuvant after three weeks. After two weeks, the last boost was injected intraperitoneally with 25 μg P48 protein in PBS buffer. Three days later, spleen cells of the immunized mice were fused with SP2/0 cells using 50% (w/v) polyethylene glycol 1000 (PEG1000) (Sigma Chemical Co., Beijing, China). Hybridoma cells were selected by HAT media (Sigma Chemical Co., Beijing, China) supplemented with 20% fetal bovine serum (FBS) (Hyclone Co., Beijing, China). The culture supernatants were screened for antibodies against P48 protein by i-ELISA as described below. Single-cell cloning was carried out three times in positive hybridomas using a limiting dilution method until monoclones were obtained.

The isotypes of the mAb were also measured by testing the culture supernatants using a commercial ELISA kit (SouthernBiotech, Birmingham, AL). Capture ELISA was performed according to the manufacturer’s protocol.

MAb 10E lgG1 was purified from mouse ascetic fluid, cultured as hybridomas in vivo and conjugated with horseradish peroxidase (HRP) (Sigma Chemical Co., Beijing, China) performed according to standard protocol [30,31]. Conjugated mAb 10E (HRP-10E) was stored with 50% glycerol at -20°C.

### I-ELISA for screening of mAb

For screening of mAb 10E, 96-well micro plates were coated with 100 ng/well of P48 protein in 0.05 M carbonate-bicarbonate buffer (pH 9.6) at 4°C overnight. After washing three times with PBS containing 0.05% Tween20 (PBST) and blocking with 200 μL 5% non-fat dried milk (Sigma Chemical Co., Beijing, China) in PBST at 37°C for 2 h, the plates were then incubated with culture supernatant at 37°C for 1 h, and washed three times with PBST. HRP-conjugated goat-anti-mouse lgG (Kpl, Gaithersburg, TN), diluted 5,000 times, were incubated at 37°C for 1 h and then washed with PBST four times. Substrate 3, 3′, 5, 5′-tetramethylbenzidine (TMB) was added to each well for visualization for at 37°C 10 min, and then 50 μL/well of 2 M sulfuric acid was added to stop the reaction was stopped. The optical density was read at 450 nm [32] in a micro plate reader (Bio-Rad, Hercules, USA).

### Cross-reactivity and specificity of mAb 10E

Western blot was used to measure the cross-reactivity of mAb 10E. Twenty micrograms of whole cell lysates of *M. bovis*, *M. bovirhinis*, *M. arginine*, *M. agalactiae*, IBRV, BADV3, BPIV3, and BVDV were analyzed by 12% SDS-PAGE, transferred to PVDF membranes, and blocked with 5% non-fat dried milk at room temperature for 4 h on a rocker. After incubation with 1:5,000 purified mAb 10E at 4°C overnight, the membranes were washed three times with PBST and incubated with 1:5,000 HRP-conjugated goat-anti-mouse lgG at room temperature for 30 min. Then washed the membranes four times with PBST, added ELC plus substrate (Applygen, Beijing, China), placed x-ray film on the top of the membranes, and exposed in dark room with safe light.

### Establishment of Dc-ELISA

Micro plates (96-well) were coated with approximately 400 ng/well purified P48 protein in 0.05 M carbonate-bicarbonate buffer (pH 9.6) at 4°C overnight and washed with PBST and blocked with 200 μl 10% horse serum at 37°C for 2 h. Fifty microlitre non-diluted bovine serum (two wells were added 50 μL PBST as mAb control) and 50 μL 1:250 mAb HRP-10E were added. After incubation at 37°C for 1 h, all unbound materials were removed by washing with PBST four times. The colorimetric reaction was achieved as described above. The percent inhibition (PI) values were determined using the formula: PI (%) = (1- OD_450nm_ of test serum/OD_450nm_ of monoclonal control) × 100%. Twenty negative bovine sera were adopted to determine the PI cut-off value, which was designed as the mean PI of 20 negative sera + 2 standard deviations (SD), which would ensure that 95% of PI values for the negative sera fall within this range.

### Dot blot assay

Dot blot assay was carried out in this study to detect the antibody to *M. bovis* and compare these data with Dc-ELISA. Briefly, *M. bovis* whole cell lysates (20 μg) and PBS (negative control) were spotted onto nitrocellulose membranes and allowed to air dry. Then membranes were blocked with 10% goat sera in DB buffer (20 mM Tris, 0.5 M NaCl, 0.055% Tween80, pH 7.5) at room temperature for 4 h and incubated with 1:400 bovine sera at 4°C overnight. After washing three times with DB buffer, the membranes were incubated with 1:5,000 HRP-conjugated goat-anti-bovine IgG at room temperature for 30 min, and then washed the membrane four times with DB buffer, added ELC plus substrate, placed x-ray film on top of membranes, and exposed in dark room with safe light.

### Comparisons of Dc-ELISA, two commercial i-ELISA kits, and Dot blot

A total of 165 bovine serum samples from five farms in different provinces of China were detected using Dc-ELISA, two commercial i-ELISA kits from Bio-X (Jemelle, Belgium) and Biovet (Saint-Hyacinthe, Canada), and a Dot blot assay. The i-ELISAs were performed as recommended by the manufacturer.

### Statistical analysis

SPSS v19.0 software was used to analyze the normality test of of the negative bovine sera PI values using a Shapiro-Wilk test and the degrees of agreement between Dc-ELISA and two commercial kits, and Dot blot by kappa statistics.

## Competing interests

The authors declare that they have no competing interests.

## Authors’ contributions

PF and WXW took the lead on the assay development and initial testing. PF and ZQY designed the evaluation procedure and ZHS performed the evaluation of the test performance. PF and ZQY carried out the field evaluation. PF and YWZ developed the result evaluation. PF and WXW wrote the manuscript. JF, HYZ and DS provided critical feedback on the manuscript. All authors read and approved the final manuscript.

## References

[B1] Nicholas R Ayling R Mycoplasma bovis: disease, diagnosis, and control Res Vet Sci 2003 74 2 105 10.1016/S0034-5288(02)00155-8 12589733

[B2] Xin J Li Y Guo D Song N Hu S Chen C Pei J Cao P First isolation of Mycoplasma bovis from calf lung with pneumoniae in China Chin J Prev Vet Med 2008 30 9 661 664

[B3] Qi J Guo A Cui P Chen Y Mustafa R Ba X Hu C Bai Z Chen X Shi L Comparative geno-plasticity analysis of Mycoplasma bovis HB0801 (Chinese isolate) PLoS ONE 2012 7 8239 10.1371/journal.pone.0038239 PMC3365025 22693604

[B4] Dénes B Tenk M Tekes L Varga I Ferenczné IP Stipkovits L Recognition of multiple Mycoplasma bovis antigens by monoclonal antibodies Hybrid Hybridomics 2003 22 1 11 16 10.1089/153685903321538035 12713685

[B5] Ghadersohi A Fayazi Z Hirst R Development of a monoclonal blocking ELISA for the detection of antibody to Mycoplasma bovis in dairy cattle and comparison to detection by PCR Vet Immunol Immunopathol 2005 104 3–4 183 15734539 10.1016/j.vetimm.2004.11.008

[B6] Sachse K Helbig J Lysnyansky I Grajetzki C Müller W Jacobs E Yogev D Epitope mapping of immunogenic and adhesive structures in repetitive domains of Mycoplasma bovis variable surface lipoproteins Infect Immun 2000 68 2 680 687 10.1128/IAI.68.2.680-687.2000 10639433 PMC97192

[B7] Rosati S Pozzi S Robino P Montinaro B Conti A Fadda M Pittau M P48 major surface antigen of Mycoplasma agalactiae is homologous to a malp product of Mycoplasma fermentans and belongs to a selected family of bacterial lipoproteins Infect Immun 1999 67 11 6213 6216 10531294 10.1128/iai.67.11.6213-6216.1999 PMC97020

[B8] Robino P Alberti A Pittau M Chessa B Miciletta M Nebbia P Le Grand D Rosati S Genetic and antigenic characterization of the surface lipoprotein P48 of Mycoplasma bovis Vet Microbiol 2005 109 3–4 201 15985342 10.1016/j.vetmic.2005.05.007

[B9] Nicholas R Baker S Recovery of mycoplasmas from animals In Mycoplasma Protocols Methods 1998 Springer 37 43 10.1385/0-89603-525-5:37 9711638

[B10] Ball H Finlay D Reilly G Sandwich ELISA detection of Mycoplasma bovis in pneumonic calf lungs and nasal swabs Vet Rec 1994 135 22 531 532 10.1136/vr.135.22.531 7879368

[B11] Bell C Blackburn P Patterson I Ellison S Ball H Real-time PCR demonstrates a higher prevalence of Mycoplasma bovis in Northern Ireland compared with sandwich ELISA Vet Rec 2012 171 16 402 22915678 10.1136/vr.100905

[B12] Ayling R Nicholas R Johansson K Application of the polymerase chain reaction for the routine identification of Mycoplasma bovis Vet Rec 1997 141 12 307 308 10.1136/vr.141.12.307 9330476

[B13] Cremonesi P Vimercati C Pisoni G Perez G Ribera AM Castiglioni B Luzzana M Ruffo G Moroni P Development of DNA extraction and PCR amplification protocols for detection of Mycoplasma bovis directly from milk samples Vet Res Commun 2007 31 225 227 10.1007/s11259-007-0011-x 17682881

[B14] Hotzel H Sachase K Pfützner H Rapid detection of Mycoplasma bovis in milk samples and nasal swabs using the polymerase chain reaction J Appl Microbiol 1996 80 5 505 510 10.1111/j.1365-2672.1996.tb03249.x 9072522

[B15] Pinnow C Butler J Sachse K Hotzel H Timms L Rosenbusch R Detection of Mycoplasma bovis in preservative-treated field milk samples J Dairy Sci 2001 84 7 1640 10.3168/jds.S0022-0302(01)74599-7 11467814

[B16] Björn J Hermeyer K Jechlinger W Zimmermann M Spergser J Rosengarten R Marion H-T *In situ* hybridization for the detection of Mycoplasma bovis in paraffin-embedded lung tissue from experimentally infected calves J Vet Diagn Investig 2010 22 1 90 93 10.1177/104063871002200117 20093691

[B17] Hermeyer K Peters M Brügmann M Jacobsen B Hewicker-Trautwein M Demonstration of Mycoplasma bovis by immunohistochemistry and *in situ* hybridization in an aborted bovine fetus and neonatal calf J Vet Diagn Investig 2012 24 2 364 369 10.1177/1040638711435145 22362536

[B18] Byrne W McCormack R Ball H Brice N Baker S Ayling R Nicholas R Application of an indirect ELISA to milk samples to identify cows with Mycoplasma bovis mastitis Vet Rec 2000 146 13 368 369 10.1136/vr.146.13.368 10803981

[B19] Le Grand D Bézille P Calavas D Poumarat F Brank M Citti C Rosengarten R Serological prevalence of Mycoplasma bovis infection in suckling beef cattle in France Vet Rec 2002 150 9 268 273 10.1136/vr.150.9.268 11918048

[B20] Caswell JL Archambault M Mycoplasma bovis pneumonia in cattle Anim Health Res Rev 2008 8 2 161 18218159 10.1017/S1466252307001351

[B21] Perez Casal J Prysliak T Detection of antibodies against the Mycoplasma bovis glyceraldehyde-3-phosphate dehydrogenase protein in beef cattle Microb Pathog 2007 43 5–6 189 17689221 10.1016/j.micpath.2007.05.009

[B22] Reddington J Reddington G MacLachlan N A competitive ELISA for detection of antibodies to the group antigen of bluetongue virus J Vet Diagn Investig 1991 3 2 144 147 10.1177/104063879100300207 1716467

[B23] Hirota J Shimizu S A new competitive ELISA detects West Nile virus infection using monoclonal antibodies against the precursor-membrane protein of West Nile virus J Virol Methods 2013 188 1-2 132 138 10.1016/j.jviromet.2012.12.002 23266257

[B24] Sharma N Hotta A Yamamoto Y Fujita O Uda A Morikawa S Yamada A Tanabayashi K Detection of Francisella tularensis-specific antibodies in patients with tularemia by a novel competitive enzyme-linked immunosorbent assay Clin Vaccine Immunol 2013 20 1 9 16 10.1128/CVI.00516-12 23114700 PMC3535769

[B25] Dufour-Gesbert F Kempf I Kobisch M Development of a blocking enzyme-linked immunosorbent assay for detection of turkey antibodies to Mycoplasma meleagridis Vet Microbiol 2001 78 3 275 284 10.1016/S0378-1135(00)00280-7 11165071

[B26] Ferrin NH Fang Y Johnson CR Murtaugh MP Polson DD Torremorell M Gramer ML Nelson EA Validation of a blocking enzyme-linked immunosorbent assay for detection of antibodies against porcine reproductive and respiratory syndrome virus Clin Diagn Lab Immunol 2004 11 3 503 514 15138175 10.1128/CDLI.11.3.503-514.2004 PMC404579

[B27] Schunk MK Macallum GE Applications and optimization of immunization procedures ILAR J 2005 46 3 241 257 10.1093/ilar.46.3.241 15953832

[B28] Villarroel A Regalado M A fast and simple method to introduce multiple distant point mutations Tech Tips Online 1997 2 1 24 26 10.1016/S1366-2120(08)70021-7

[B29] Bradford MM A rapid and sensitive method for the quantitation of microgram quantities of protein utilizing the principle of protein-dye binding Anal Biochem 1976 72 1 248 254 942051 10.1016/0003-2697(76)90527-3

[B30] Wilson M Recent development in the periodate method of conjugating horseradish peroxidase (HRPO) to antibodies Immunofluorescence and Related Staining Techniques 1978 215 224

[B31] Hendriksen C De Leeuw W Production of monoclonal antibodies by the ascites method in laboratory animals Res Immunol 1998 149 6 535 542 10.1016/S0923-2494(98)80002-3 9835414

[B32] Chusri M Wongphanit P Palaga T Puthong S Sooksai S Komolpis K Production and characterization of a monoclonal antibody against enrofloxacin J Microbiol Biotechnol 2013 23 1 69 75 10.4014/jmb.1201.01017 23314370

